# Older Adult Health and Remission of Risky Drinking and Alcohol Use Disorder: A Lifespan-Developmental Study

**DOI:** 10.1093/geroni/igab046.1114

**Published:** 2021-12-17

**Authors:** Matthew Lee, Ellen Yeung, Thomas Kwan, Kenneth Sher

**Affiliations:** 1 Rutgers University, Piscataway, New Jersey, United States; 2 George Washington University, Washington, District of Columbia, United States; 3 University of Missouri, Columbia, Columbia, Missouri, United States

## Abstract

Older-adult drinking is a growing public-health concern. As part of a larger project investigating older adulthood by contrasting this with other adult developmental periods, this study used longitudinal U.S.-representative data to test bidirectional associations between drinking and health, emphasizing aging-related health concerns as potential mechanisms of remission from risky/problem drinking. In multiple-group cross-lag models, we found that effects of poor self-reported health on drinking reductions increased with age, reached significance around midlife, and were strongest in older adulthood. However, a caveat revealed by additional Markov transition models was that these effects did not extend to relatively severe older-adult drinkers (indexed by DSM-5 AUD). In some instances, poor health even predicted less older-adult AUD remission. Altogether, findings support the notion of aging-related health concerns as important mechanisms of older-adult drinking reduction; but highlight a need to understand barriers to these mechanisms among severe older-adult drinkers, in part toward guiding lifespan-developmentally-informed interventions.

